# Fundamental Understanding of Cellular Water Transport Process in Bio-Food Material during Drying

**DOI:** 10.1038/s41598-018-33159-7

**Published:** 2018-10-12

**Authors:** Md. Imran H. Khan, Troy Farrell, S. A. Nagy, M. A. Karim

**Affiliations:** 1 Science and Engineering Faculty, Queensland University of Technology (QUT), Brisbane, Queensland Australia; 2 Department of Mechanical Engineering, Dhaka University of Engineering & Technology, Gazipur, Gazipur-1700, Bangladesh; 3 MTA-PTE, Clinical Neuroscience MR Research Group, Pécs, Hungary; 4 Pécs Diagnostic Centre, Pécs, Hungary; 5 Department of Neurosurgery, University of Pécs, Medical School, Pécs, Hungary

## Abstract

Bio-food materials are heterogeneous in structure with cellular diversity, where the majority of the water is located in the intracellular spaces. Understanding of the nature of the microscopic behaviour of water transport is crucial to enhance the energy efficiency in food processing and obtain the better quality of processed food. In this research, apoplastic and symplastic transport of cellular water in the bio-food material during drying was investigated using ^1^H-NMR-T_2_ relaxometry. We found that intracellular water (ICW) migrates from intracellular spaces to the intercellular spaces by progressive rupturing the cell membranes while drying at a higher temperatures (60 °C–70 °C). In this case, apoplastic process dominates the transport process. However, at lower temperature (45 °C), cell membranes do not rupture and therefore ICW migrates from cell to the neighbouring cell through micro-capillaries, where the symplastic process dominates the mass transfer at different stages of drying.

## Introduction

**Figure 1:**
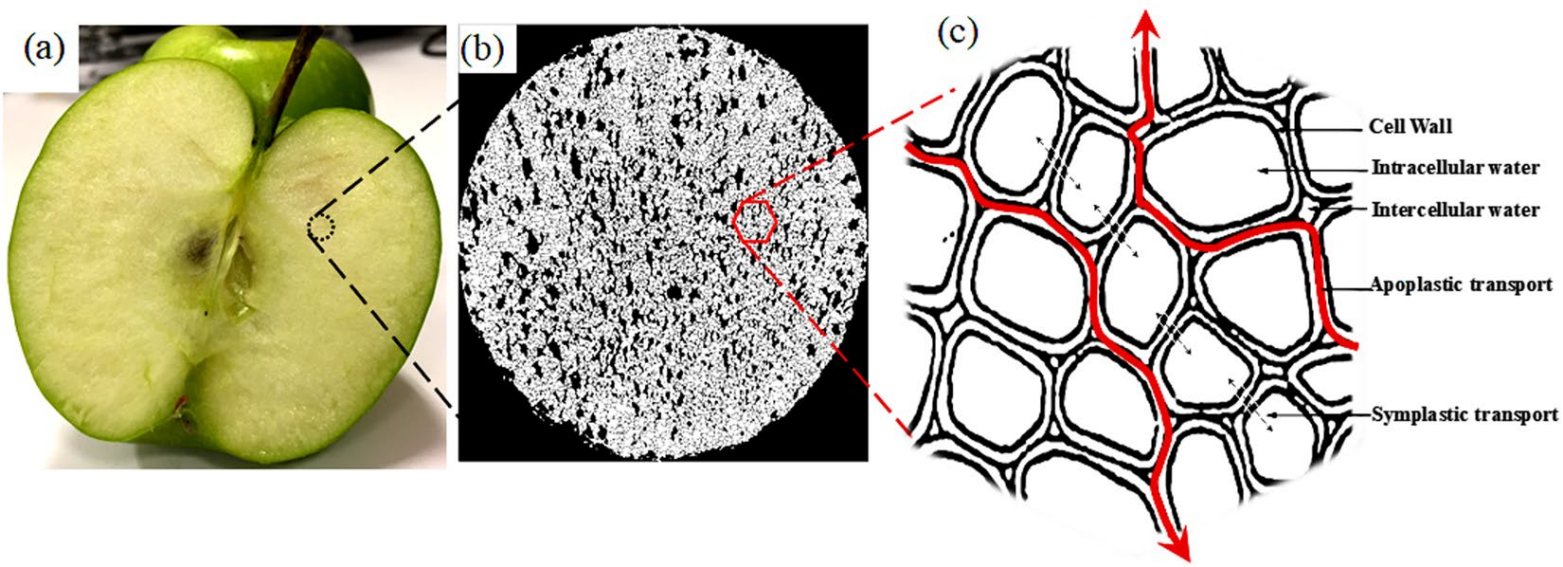
Cellular water transport process in apple tissue.

Postharvest loss is a major concern for food security as about one-third of global food is lost due to the lack of proper processing or preservations^1^. Dying is the dominant food preservation method^2^, which has a great potential to significantly reduce the postharvest losses, if properly designed and used. However, the structural heterogeneity of bio-food material makes it complex to understand the micro level transport process and morphological changes that occur during drying. Bio-food materials are mostly porous in nature with cellular structure (heterogeneous) and contains approximately 80–90% water in the different cellular environments, namely intercellular environment, intracellular environment, and cell wall environment each having different proportions^3,4^. Intercellular spaces or environments are the spaces between the cells as shown in Fig. 1. They are mostly composed of air, a small portion of water, and other solutes (such as sugar)^4^. This water is commonly referred as free water (FW) as it can be easily removed during drying. Within the cell, the vacuole and cytoplasm together act as water reservoirs in the food tissue. This water is known as intracellular water (ICW)^4^ and also sometimes referred as loosely bound water (LBW)^5^. In bio-food material, 78–95% of total water is found in intracellular spaces^5^. Water that is integrated with cell wall structure accounts for 2–5% of total water and is referred as tightly bound water (STBW)^5^ as it cannot be easily removed during drying.

This structural heterogeneity makes the bio-food material complex to understand the morphological behaviour during drying although considerable efforts have been made in this regard. Most of the previous studies have been conducted at the macroscopic tissue level^6–8^. These studies consider the bio-food material to be homogenous and hence it does not differentiate the properties of pores, cell walls, cell membranes and cell vacuoles^9,10^. Such simplistic assumptions do not provide a detailed, cell level, mechanistic insight into the physical processes, such as cracking or shrinking, which occur because of drying^11^. This deficiency in understanding of drying process has motivated some researchers to investigate transport processes and morphological changes at a microscopic level. Khan *et al*.^12,13^ developed a conceptual understanding of cellular water transport phenomena during drying. However, they did not investigate the effect of microstructural changes on transport phenomena. Therefore, fundamental understanding of cellular water migration mechanism remains unclear. Moreover, it is well known that the drying temperature strongly affects transport process^14^ and significantly modify the drying kinetics^15^. However, the existing literature does not report the effect of temperature on cellular level water transport. Some theoretical works on micro level mass transfer have been proposed^16–19^. However, these models considered oversimplified assumptions due to the lack of fundamental understanding of the micro-level water transport mechanisms.

In bio-food materials, microscopic water transport follows different cellular pathways during drying. FW migrates from intercellular spaces to the surface of the sample through diffusion and then is removed by evaporation to the drying medium^20^. Transport of ICW is quite complex. Two types of transports, namely, symplastic transport and apoplastic transport are thought to take place. Symplastic transport occurs when water is travelled between two neighbouring cells through microcapillaries in the cell walls, as shown in Fig. 1. The transport pathway of this process is often referred as intracellular pathway^21^. Generally, it is assumed that simple Fickian diffusion drives the symplastic transport^14–17^. Apoplastic transport occurs when ICW migrates from the cell to the intercellular spaces (Fig. 1). This pathway can be defined as the extracellular pathway. The physical driving mechanism for ICW transport in apoplastic transfer during drying is unclear. Due to this significant research gap, existing microscale models have been formulated considering only the symplastic transport process, which make the models oversimplified. Understanding of the physical mechanism of apoplastic transport of ICW during drying of bio-food material is the necessity for next generation food drying research. Therefore, the main aim of this paper is to uncover the ICW transport process, and evaluate the effect of different drying temperature on apoplastic transport process during drying.

Several methods, including differential scanning calorimetry (DSC), Dilatometry (DIL), bioelectric impedance analysis (BIA), nuclear magnetic resonance (NMR), and X-ray microtomography have been used to analyse the cellular level water in different biological food tissues. However, most of these methods have some specific limitations. For instance, tissue geometry strongly affects the result during the BIA experiments, which ultimately affects the validity of the BIA method. It is not possible to use the DSC with a hot air drying setup as DSC can only work at or below the freezing temperature of water^22^. Due to the limitations of the above methods, except NMR and X-ray microtomography, they are not suitable for the application in plant-based food tissue during drying for measuring the cellular level water^22^. Therefore, in this study, this two techniques (NMR and X-ray microtomography) will be extensively used to investigate the ICW transport process.

Nuclear magnetic resonance (NMR) T_2_ relaxometry will be used to investigate cellular water transport mechanism, as this method was previously successfully used to investigate the cellular water distribution in different biological tissue such as animal lung^23,24^, brain^25–29^, liver^30,31^, and plant tissue^5,32^.

In this study, granny smith apple will be used as a bio-food material where the water protons dominate the ^1^H-NMR proton signals, which are an average over the whole sample that provides the information about the water content in the biological tissue^33^. It is established that the proton density inside the tissue of the sample is directly proportional to the NMR signal intensity^34^. This signal intensity can be characterised by the nature of spin-spin (T_2_) relaxation which is the transverse component of the magnetization vector. From the T_2_ relaxation analysis, it is straightforward to see the number of decays in the relaxation curve, and therefore it is possible to investigate different cellular level water based on their intensity of relaxation. However, the low sensitivity is the main concern of NMR analysis which causes excessive noisy data. Therefore, the NMR results need to be justified or cross checked by other methods. X-ray micro tomographic image analysis is a viable method to physically observe the morphological changes in animal tissues^35–38^ as well as different plant-based food tissues such as apple^39,40^, banana^41^, and mango^42^. In X-ray micro tomography, the image segmentation and filtration algorithms are used to investigate the liquid or solid phase inside the X-ray image^43^. The detail explanation of X-ray microtomography and NMR methods has been presented in methodology section (section 4).

## Results and Discussion

**Figure 2:**
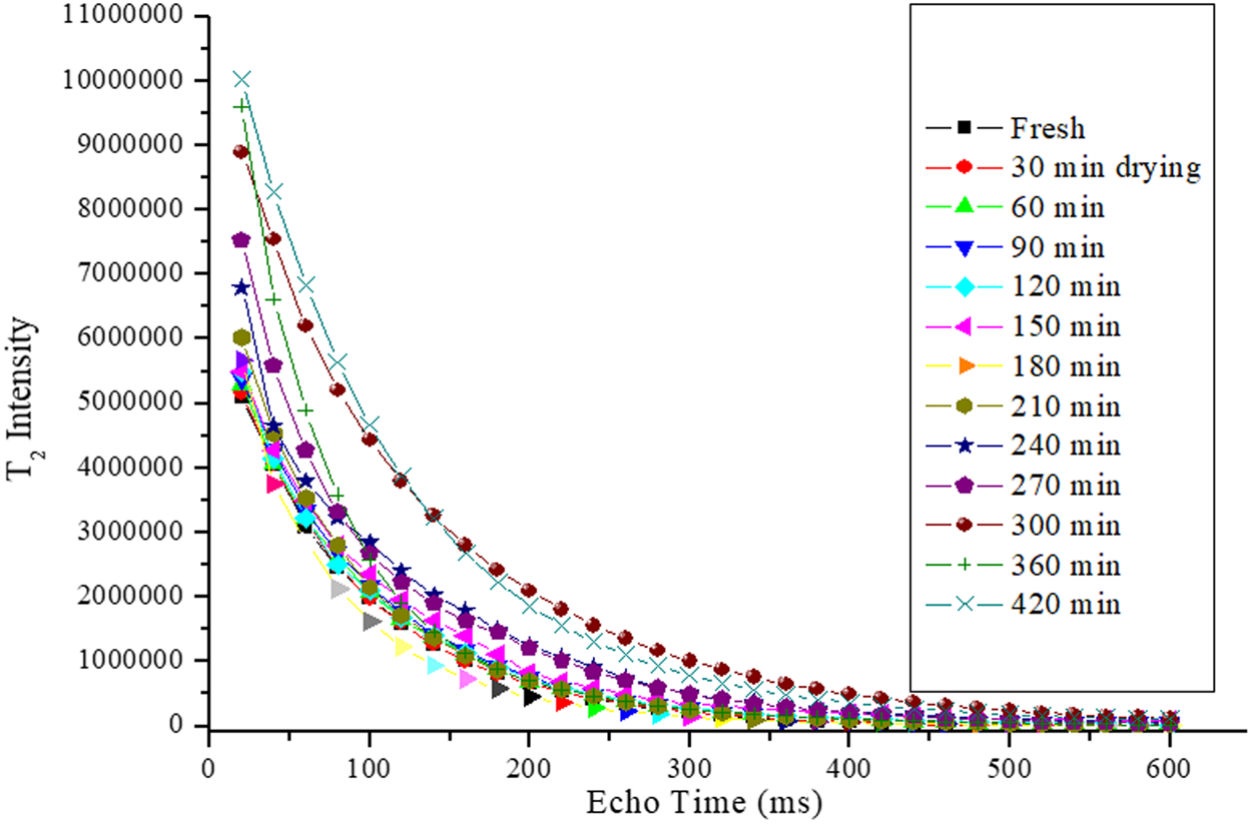
T_2_ intensity data at different stages of drying.

^1^H-NMR-T_2_ relaxometry was used to measure the proportion of intracellular water present in apple tissue at different stages of drying. Figure 2 shows the T_2_ relaxation decay curve obtained from samples of drying at various drying times. The T_2_ data shown in Fig. 2 was obtained while the sample was dried at a constant drying temperature of 60 °C. By fitting the bi-exponential decay Eq. (1) with these different T_2_ relaxation intensity data, two different water proton relaxations (long and short) were calculated and presented in Table 1. Based on the mobility of different cellular water, pore size, membrane permeability, and the proton density within the sample, these two (long and short) components are categorised as ICW and FW, respectively. The proportion of ICW and FW were then calculated from the relative contribution of long and short T_2_ components. The detail procedure that developed by the current authors to identify the ICW and FW using ^1^H-NMR-T_2_ relaxometry can be found in authors’ pevious publication^5^.

**Table 1:** Different T_2_ component at different drying times, each measurement is the mean and standard deviation (SD) of 3 tests.

Drying Temperature	Drying time (min)	Long component		Short component		Goodness of fit (R^2^)
		T_2_ (ms)	%	T_2_ (ms)	%	
45 °C	Fresh	90.67 ± 11.3	88.10 ± 7.9	32.76 ± 4.8	11.97 ± 3.8	0.9998
	30	92.43 ± 7.5	87.96 ± 6.4	35.55 ± 6.9	12.04 ± 4.9	0.9788
	60	96.52 ± 13.7	82.52 ± 7.6	34.86 ± 5.6	13.88 ± 5.3	0.9887
	90	89.25 ± 14.4	78.14 ± 6.3	48.21 ± 12.5	17.48 ± 4.9	0.9985
	120	88.74 ± 11.9	78.81 ± 10.5	29.24 ± 5.8	21.86 ± 6.2	0.9956
	150	90.12 ± 9.8	79.22 ± 7.3	26.27 ± 4.9	21.19 ± 3.8	0.9785
	180	100.19 ± 13.8	82.13 ± 5.8	25.88 ± 5.2	20.78 ± 6.5	0.9878
	210	125 ± 15.9	81.06 ± 8.9	18.98 ± 8.9	17.87 ± 4.7	0.9988
	240	100.12 ± 10.5	79.80 ± 7.6	16.97 ± 3.8	18.94 ± 6.3	0.9948
	270	108.72 ± 13.7	82.01 ± 9.4	28.27 ± 7.8	20.20 ± 7.1	0.9995
	300	106.92 ± 10.2	82.71 ± 6.7	19.28 ± 9.4	17.99 ± 6.2	0.9758
	360	98.86 ± 16.9	84.23 ± 8.4	20.52 ± 7.5	15.77 ± 7.1	0.9855
	420	182.56 ± 11.7	84.51 ± 9.3	13.58 ± 8.5	15.49 ± 6.4	0.9769
60 °C	30	90.38 ± 10.4	87.32 ± 9.5	36.85 ± 6.2	12.68 ± 4.2	0.9989
	60	99.44 ± 8.5	78.30 ± 8.4	32.56 ± 4.3	21.7 ± 4.9	0.9997
	90	116.83 ± 11.7	45.10 ± 7.6	68.51 ± 8.7	54.9 ± 3.8	0.9999
	120	102.93 ± 11.3	69.41 ± 6.8	28.14 ± 6.4	30.59 ± 4.9	0.9998
	150	109.54 ± 13.7	78.45 ± 8.8	25.13 ± 5.3	21.55 ± 4.8	0.9996
	180	76.74 ± 13.4	53.46 ± 9.6	12.10 ± 4.7	46.54 ± 7.1	0.9979
	210	98.42 ± 11.7	65.03 ± 6.4	43.18 ± 6.8	34.97 ± 4.8	0.9984
	240	123.51 ± 14.4	74.45 ± 6.9	8.95 ± 4.4	25.55 ± 5.7	0.998
	270	125.18 ± 12.5	51.72 ± 5.7	31.02 ± 8.9	48.28 ± 6.9	0.9987
	300	141.48 ± 14.3	76.93 ± 6.9	50.12 ± 10.6	23.07 ± 4.5	0.9998
	360	121.05 ± 11.4	20.02 ± 4.5	47.61 ± 9.7	79.98 ± 11.2	0.9988
	420	284.98 ± 17.8	5.12 ± 3.7	99.89 ± 11.7	94.88 ± 13.4	0.9896
70 °C	30	109.34 ± 10.2	81.59 ± 10.9	61.89 ± 5.6	18.41 ± 6.4	0.999.7
	40	133.12 ± 9.3	58.02 ± 8.9	27.45 ± 7.2	41.98 ± 6.2	0.9986
	60	120.42 ± 6.5	42.86 ± 7.5	20.58 ± 6.3	57.14 ± 9.1	0.9978
	90	148.08 ± 12.8	70.59 ± 6.4	34.35 ± 7.5	29.41 ± 5.7	0.9988
	120	137.30 ± 10.7	46.12 ± 6.5	32.50 ± 4.9	53.88 ± 9.8	0.9986
	150	120.82 ± 12.2	60.03 ± 7.8	28.12 ± 6.1	39.97 ± 3.9	0.9975
	180	98.58 ± 12.9	78.26 ± 9.7	21.11 ± 5.7	21.74 ± 5.7	0.9987
	210	95.26 ± 10.8	60.07 ± 8.5	33.28 ± 7.2	39.93 ± 8.1	0.9998
	240	128.21 ± 15.3	81.50 ± 7.5	18.25 ± 6.2	18.50 ± 3.7	0.9992
	270	139.12 ± 11.8	30.10 ± 7.9	35.13 ± 7.9	69.90 ± 6.9	0.9988
	300	137.38 ± 13.7	10.11 ± 4.6	56.22 ± 9.9	89.89 ± 11.3	0.9998
	360	119.55 ± 12.5	4.20 ± 3.1	49.21 ± 8.6	95.80 ± 11.9	0.9895
	420	284.87 ± 22.2	3.50 ± 2.5	95.79 ± 12.6	96.50 ± 13.2	0.9885

### ICW transport mechanism at different drying conditions

**Figure 3:**
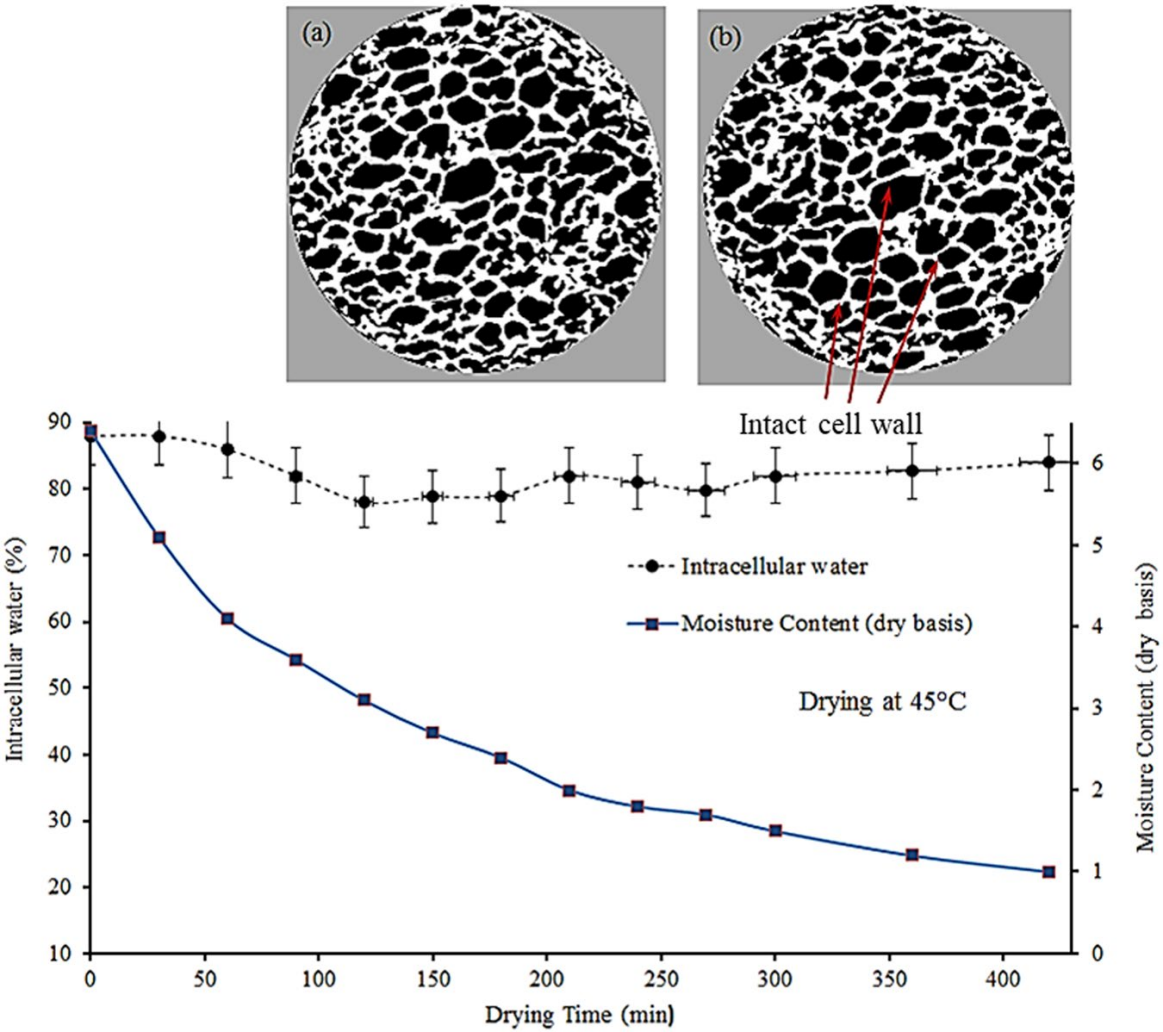
Transport of intracellular water during drying of apple tissue at 45 °C, including X-ray tomographic image of the dried sample (**a**) after 260 mins of drying), (**d**) after 360 mins of drying.

**Figure 4:**
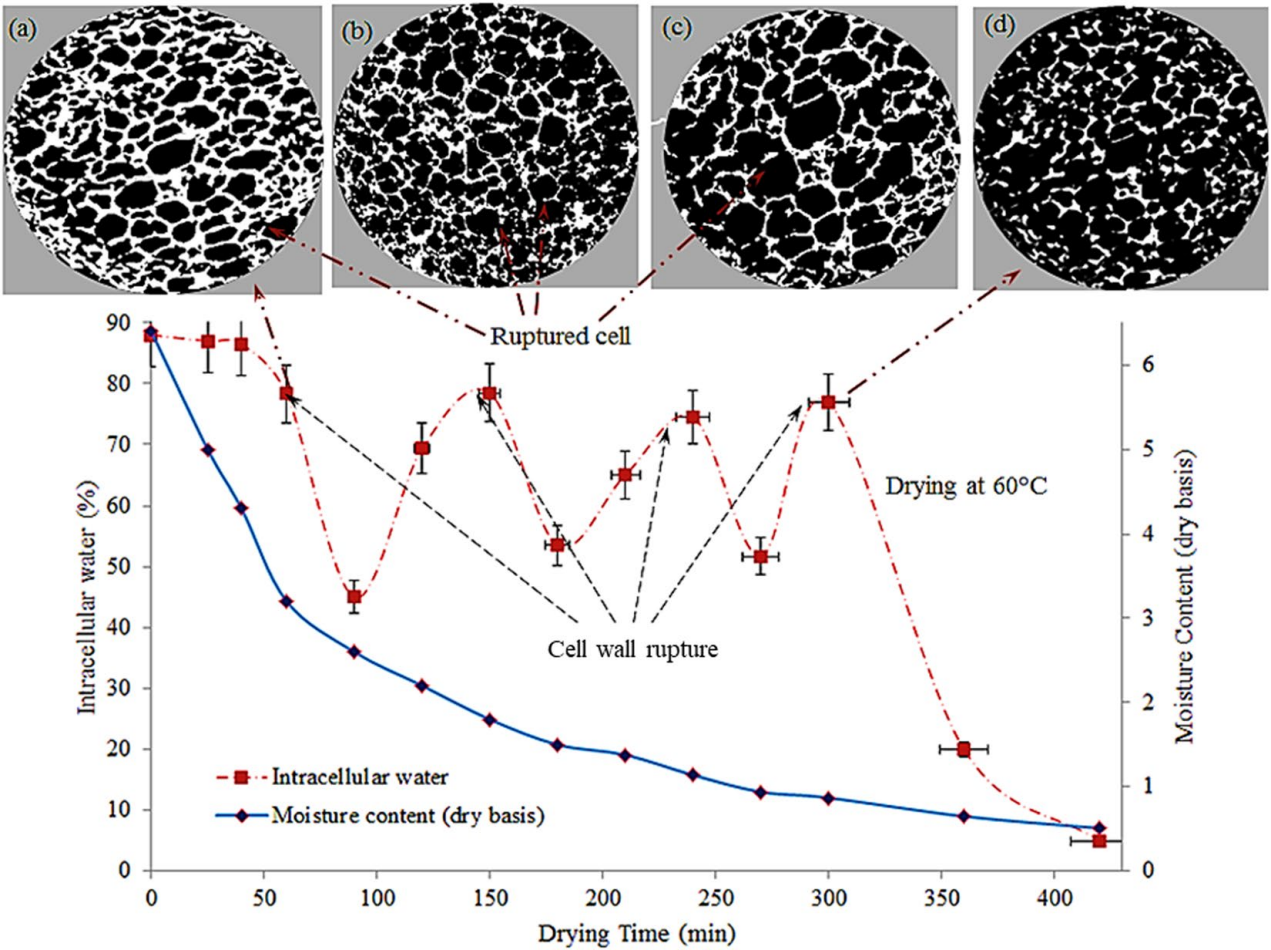
Transport of intracellular water during drying of apple tissue at 60 °C, including X-ray tomographic image of the dried sample (**a**) after 70 mins of drying), (**b**) after 150 mins of drying, (**c**) after 250 mins of drying, and (**d**) after 300 mins of drying.

**Figure 5:**
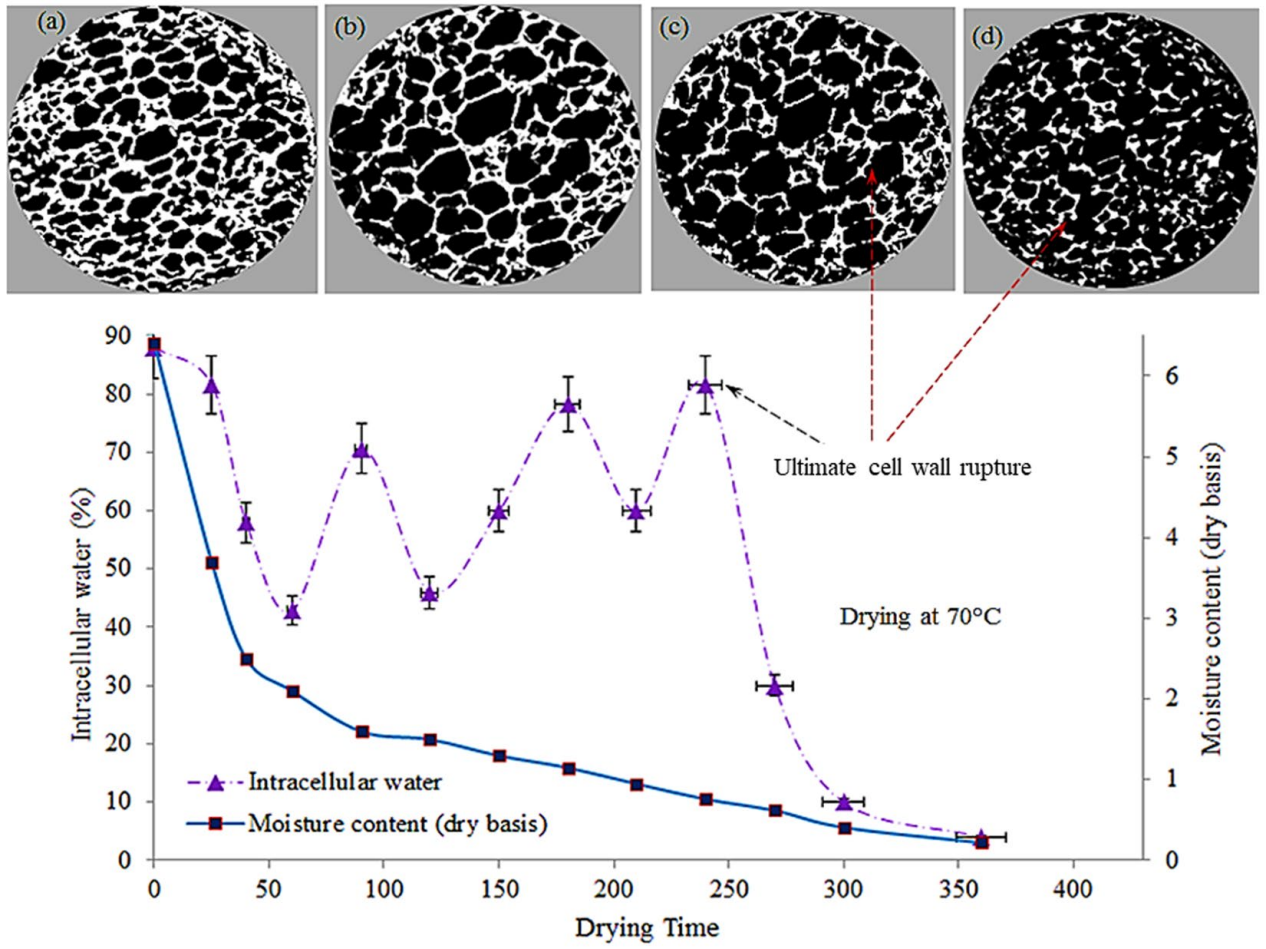
Transport of intracellular water during drying of apple tissue at 70 °C, including X-ray tomographic image of the dried sample (**a**) after 40 mins of drying), (**b**) after 150 mins of drying, (**c**) after 200 mins of drying, and (**d**) after 250 mins of drying.

The percentage of ICW was calculated based on the consideration that the total water (ICW + FW) at any point (instantaneous moisture) is 100%. The measured proportion of ICW was plotted with drying time as shown in Figs 3–5. As mentioned above, total water at any point is 100%. For example, in Fig. 3, ICW at 120 minutes (fifth point in the graph) is 88% and therefore rest 12% is FW.

It can be seen in the Figs 4–5, there are many fluctuations in the proportion of ICW and FW throughout the drying. These fluctuations are mainly caused by the cell collapse at different stages of drying. The peak points in the ICW curve indicate the cell membrane collapsing points. When cells collapse, water comes out of the cells, move from intracellular spaces to intercellular spaced and thus becomes FW. Therefore, when cell collapse takes place, proportion of ICW drops rapidly and proportion of FW increases accordingly as total amount of water (ICW + FW) is considered 100% at each point of the drying process. The fluctuations of ICW curve are mainly due to this progressive increase and decrease of ICW. Based on these facts, the different results at different drying conditions have been analysed and discussed.

It can be observed that during drying at low temperature (e.g. 45 °C), the spatial distribution of ICW at different stages of drying remains stable, i.e., no peaks or fluctuations (Fig. 3). This trend of the curve indicates that the cell walls did not rupture while the sample was dried at 45 °C. For physically observing the cell rupturing phenomena inside the microstructure of apple tissue, extensive micro tomographic experiments were conducted at different stages of drying. The X-ray tomographic experiments were conducted for each sample after every 60 minutes of drying and X-ray raw images were analysed to interpret the result. From the various images, two such images are presented in Fig. 3(a,b), which were taken at the final stages of drying. It can be seen from this microstructural images that the cell wall remains intact up to the end of drying. This means no cell breakage was observed during the entire drying processes.

In this case, ICW migrates from cell to the neighbouring cell and cell to the intercellular spaces through very fine capillaries, and this pathway can be defined as an intracellular pathway. Migrating ICW via intracellular pathway is a very slow process, and therefore the average moisture content decreases slowly (Fig. 3). In addition to this, a close observation of Fig. 3 reveals that there are minor fluctuations in the proportion of intracellular water content. These fluctuations may be due to the initiations of some micro cracks which cannot be clearly recognised in X-ray tomographic images. Based on the characterization of symplastic and apoplastic transport phenomena, it can be argued that symplastic process is the dominant driving mechanism for migrating ICW while drying of apple tissue at a lower temperature (e.g. 45 °C).

In drying at a higher temperature (60–70 °C), many peaks (fluctuations) were observed in the curves for the spatial distribution of ICW at different stages of drying, as shown in Figs 4 and 5. These different peaks indicate the cell rupturing point where cell walls were ruptured at different stages of drying, resulting ICW migration from the intracellular environment to the intercellular environment via extracellular pathways. Although a small portion of ICW transports via intracellular pathways, apoplastic transport is the dominant driving mechanism for migrating ICW while drying at a higher temperature (60–70 °C) as most of the ICW migrates through extracellular pathways after rupturing the cell walls.

**Figure 6:**
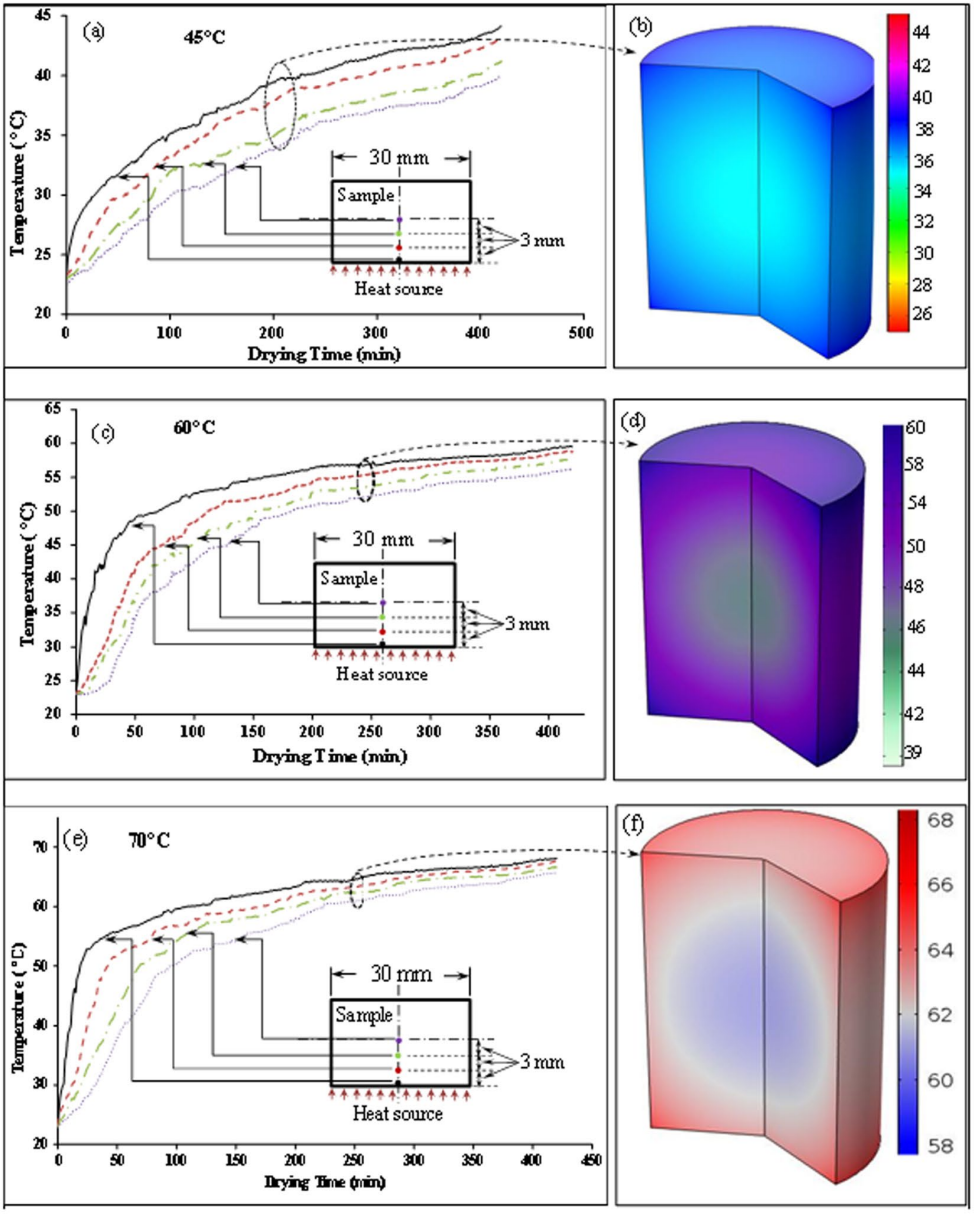
Spatial distribution of Temperature during drying of apple tissue (**a**,**b**) experimental and simulated result at 45 °C, (**c**,**d**) experimental and simulated result at 60 °C, and (**e**,**f**) experimental and simulated result at 70 °C.

At the first stage of drying, the ICW remains constant up to 50 mins of drying, as shown in Fig. 4. During this time, mostly FW migrates to the surroundings through evaporation. When most of the free water is transported to the air, the temperature of the surface and adjacent cells starts to rise and thermal and mechanical stress are developed on the cells and eventually results in the rupture of the cells. Consequently, the first stage cell rupture occurred after 60 mins of drying and hence ICW curve declined rapidly and dropped to about 42% as shown in Fig. 4. After rupture of the cell walls, the ICW travels quickly to the intercellular spaces via extracellular pathways without facing any barrier (from cell membrane as these are now broken) to transport. The migrated ICW then become FW which is then transported to the surrounding through evaporation. Due to this evaporation, amount of FW again decreases, and the relative proportion of ICW increases, as shown in Fig. 4 from 90 minutes to 150 minutes. At this stage, temperature of internal layers is still lower than layers close to the surface and therefore a temperature gradient exists (as, shown in Fig. 6). After first stage of cell rupture, heat quickly reaches the next layer because of lower thermal resistance due to ruptures cells. It heats the cells and repeats the process of cell rupture described above.

Likewise, at the later stages of drying, the second stage cell membrane ruptures after 150 mins of drying. Consequently, again some portions of ICW are released to the intercellular environment without facing any cell membrane resistance which leads the ICW curve dropping rapidly. These consequences of cell rupture continue from the surface towards the center of the sample. The investigated cell rupturing mechanism is consistent with the authors’ previous findings^12,13^, where this cell rupturing phenomena for bound water transport was introduced for different food tissues. However, the effect of temperature on cell collapse and ICW transport were not reported previously. It is well known that the drying temperature strongly affects transport process and significantly alter the drying kinetics. However, no literature reported the effect of higher temperature on physical changes of cells and ICW transport. Therefore, this research article addressed this issue to uncover the effect of temperature on cell collapse and ICW transport. Moreover, the cell collapsing phenomena established by NMR experimental study is a new finding in food drying research. In order to further establish the findings and to physically observe evolution of the microstructure of the food tissue during drying, extensive micro tomographic experiments were conducted. Results from micro tomographic experiments (Fig. 4a–d) were in close agreement with the NMR data. It can be seen from Fig. 4a, the cell walls those are located close to the surface of the sample have been ruptured first and the cell walls towards the centre of the sample remained almost intact. This result is consistent with NMR result (first stages of cell rupture in Fig. 4). The amount of progressive rupture of cell membranes increases from surface towards centre, as shown in Fig. 4(b–d). Again, these X- ray results fully agree with the NMR results (peak points 2–4, in Fig. 4).

In addition to this water transport via cell collapsing mechanism, ICW also migrates from cell to the neighbouring cell and intercellular spaces via internal micro-capillaries as a diffusion process while cells do not experience rupturing (for instance, between the two rupturing periods, 70–140 mins of drying). Based on the definition of symplastic and apoplastic transport process, it can be argued that these two transport processes are responsible for mass transfer while drying is in progress at higher temperatures (above 60 °C). However, as most of the ICW migrates via rupturing cell membrane, therefore, apoplastic transport is the dominant mechanism for ICW transport.

During drying at 70 °C, the similar trend of cell rupturing phenomena can be observed (Fig. 5) although the cell rupture timing is changed due to the higher temperature. The rupture of the cell walls at higher temperatures mainly occurred due to the generation of thermal stresses inside the food material. The cumulative propagation of this stress towards the direction from the heating surface to the centre of the sample is responsible for the periodic rupture of cell walls. The thermal stress is mainly developed due to the temperature and moisture gradient. During drying, the heat energy is propagated from the surface towards the center of the sample. Figure 6 shows the temperature distribution and gradient inside the sample throughout the drying period at different temperatures. The temperature distribution profiles during drying at different temperatures were developed from the experimental data (Fig. 6a,c,e) as well as simulated results (Fig. 6b,d,f). The detail explanation of experimental temperature measurement and simulations can be found in materials and methods section (section 4.2).

Figure 6(a,b) shows experimental and simulated temperature distribution during drying at low temperature (45 °C). It can be observed that the surface temperature gradually increased and rose up to 44 °C, whereas, the temperature at the centre of the sample remains significantly lower than the surface temperature throughout the whole drying process. This significant temperature difference exists mainly due to the passive rate of heat transfer as no cell rupture takes place. At this temperature, the generated thermal stress is not enough to rupture the cell membrane, resulting no cell breakage was observed. As cells are not ruptured, cell walls create significant thermal resistance to heat transfer towards centre of the sample.

Figure 6(c,d) shows the spatial temperature distribution of apple tissue throughout drying at 60 °C. It can be seen that after 60 mins of drying, the surface temperature rose from room temperature to about 50 °C. The stress developed at this temperature is adequate to start the rupture of the cell membrane^12,44,45^, and therefore the cells located near the surface of the sample were ruptured first. The temperature at 3 mm from the surface towards the center of the sample was found to be 5–8 °C lower than the surface (Fig. 6c,d). Consequently, the cells located in that area remained intact. After 290 mins of drying, the whole sample temperature reached the cell collapse temperature (50 °C) and therefore the final cell collapse was observed after 300 mins of drying (Fig. 4c).

Similarly, the spatial temperature distribution in apple tissue during drying at 70 °C, is shown in Fig. 6(e,f). It can be seen that the sample surface temperature reaches the cell collapse temperature (around 50 °C) after 30 mins of drying. Consequently, the cells near the surface of the sample rupture first (Fig. 5a). The second stage of rupture occurred after 100 mins of drying. In this time, the surface temperature rose to about 58 °C and about 50 °C towards the center of the sample. As a result, progressive rupture of the cell walls was observed (Fig. 5c,d) from the surface to the center. The trends of symplastic and apoplastic transport phenomena at 70 °C are very similar to that of 60 °C throughout the drying period that are discussed above. However, there are some differences on cell rupture timing can be observed which are discussed below.

**Figure 7:**
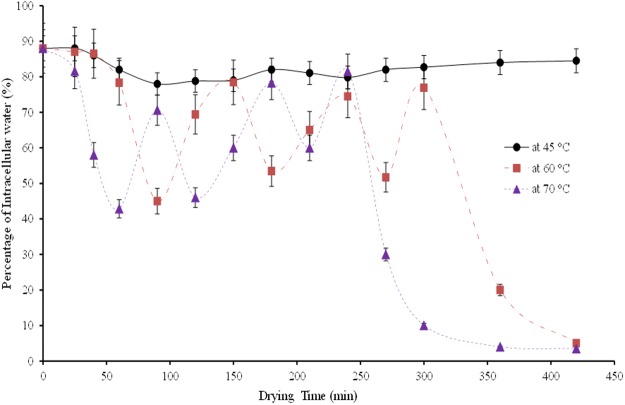
A comparison of intracellular water at different drying temperature.

A comparative analysis of ICW transport phenomena at different drying temperatures is shown in Fig. 7. At 70 °C, the cell walls start to rupture 30 mins earlier than drying at 60 °C. At the middle stages of drying, the cell wall ruptures faster at 70 °C, than drying at 60 °C. It can be observed that drying at 70 °C, the final cell wall collapse takes place after 250 mins of drying, (Fig. 5d), while the final collapse is observed after 300 mins of drying at 60 °C. These variations of cell collapsing time are mainly due to the intensity of heat energy which is more active while drying at 70 °C than 60 °C. From these analyses, it can be interpreted that drying temperature significantly affect the cell rupturing process and eventually the symplastic and apoplastic transport processes during drying.

## Summary

In this research, fundamental understanding of cellular water transport process during drying of apple tissue was uncovered. ^1^H-NMR technique was used to investigate the cellular water characteristics at different stages of drying. T_2_ relaxometry with Paravision 4 software (Bruker) was applied for the data analysis. It was found that ICW migrates from intracellular spaces to the intercellular spaces through extracellular pathways and intracellular pathways. In drying at a higher temperature (60 °C, and 70 °C), ICW is transported through both intracellular and extracellular pathways. Before reaching the cell wall collapsing temperature (50 °C) inside the sample, the ICW migrates from intracellular spaces to the intercellular spaces through micro-capillaries following the intracellular pathways. After reaching the cell wall collapsing temperature, ICW mainly migrates through rupturing cell membrane, and the pathway can be defined as extracellular pathways. This two transport pathway are driven by symplastic and apoplastic transport processes. Between this two transport processes; apoplastic transport dominates the ICW migration process when cell membranes rupturing take place. On the other hand, symplastic transport process dominates the water migration when cells remain intact. During drying at low temperature (45 °C), intracellular water transports only through intracellular pathways without rupturing the cell membranes. In this case, symplastic transport dominates the ICW migration process. The understanding of the rupturing period of cell membranes and the effect of temperature on symplastic and apoplastic transport process are crucial as this knowledge can be used for establishing a fundamental theory for cellular mass transfer inside bio-food material during drying.

## Material and Methods

### Sample Preparation and drying

Granny Smith apple as a bio-food material was taken for this study which was collected from a local market in Brisbane, Australia. Before experimenting, the samples were stored in a refrigerator at 4 °C until the drying experiments. The standard procedure for sample preparation was maintained. Before cutting the sample, the materials were appropriately cleaned to avoid any hazardous material in the sample. The washed samples cut into cylindrical slices of 30 mm length and 18 mm diameter. Drying experiments were performed using a cabinet dryer (temperature range 35–70 °C) where drying air flowed perpendicularly to the surfaces of the samples. The dryer was started 30 min prior to each drying run in order to promote a steady state drying conditions. The prepared samples were placed on the tray of the dryer and then dried at three different temperatures, namely 45 °C, 60 °C, and 70 °C. Each sample was taken from the dryer after 30 mins of drying and then immersed in an inert liquid (Fomblin PFPE) in an NMR tube. The tube with the sample was then inserted into the NMR probe. The drying experiments were performed at the above different temperatures with a constant air velocity of 0.7 m/s. The ambient air temperature was 25 °C with 60% relative humidity. During the drying process, the tray was taken out at 30 min intervals from the dryer and weighed using a digital electronic balance (model BB3000; Mettler-Toledo AG, Grefensee, Switzerland). The measurement range of the balance was 0–100 g with an accuracy of 0.001 g. The surface temperature was measured by a thermal imaging camera (Flir- i7, 140 × 140 pixels, accuracy +/−0.5%, temperature range −20 °C to 250 °C). All weighing was completed within 10 s during the drying process.

### Temperature distribution measurement

**Figure 8:**
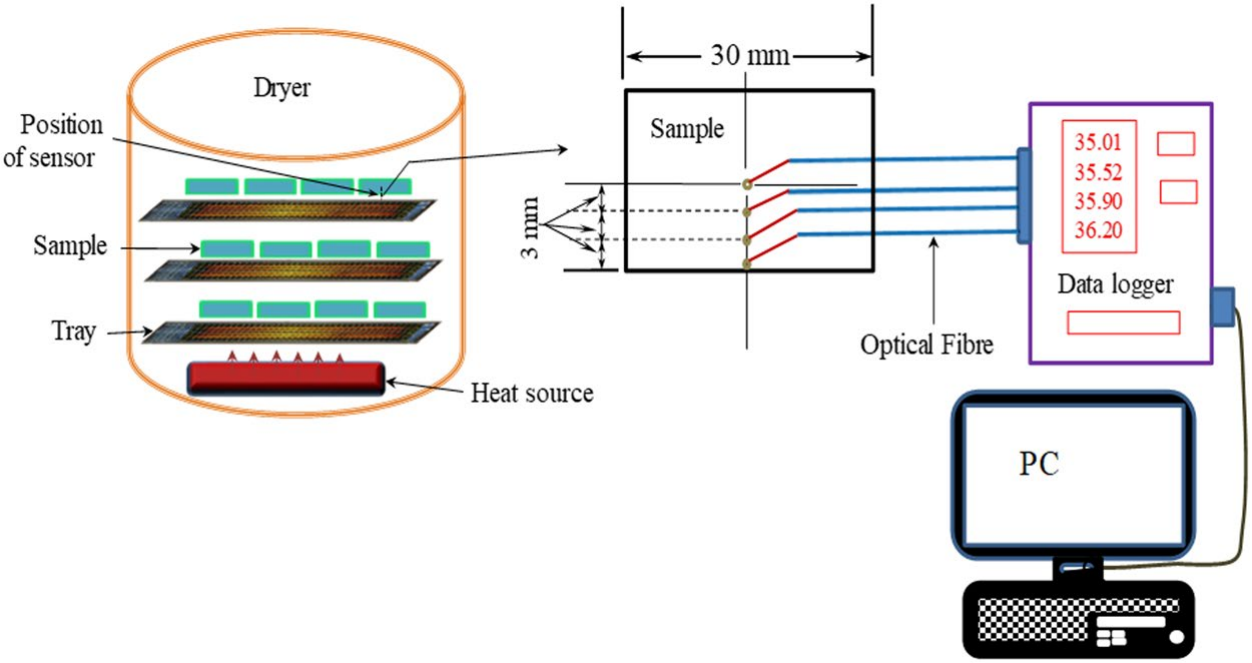
Schematic diagram for measuring temperature distribution.

In order to interpret and analyse the results, an internal temperature distribution was measured. Optical fibre thermal sensors (TS3, the flexible, temperature range −200 °C to +300 °C, fibre diameter 200 μm) were used to determine the temperature distribution inside the sample. Four optical fibre thermal sensors were placed at different locations of the samples, as shown in Fig. 8. The first sensor was placed at the centre of the sample (9 mm away from the surface), the second one was placed at 6 mm, third was placed at 3 mm away from the surface of the sample. The fourth sensor was placed on the surface of the sample (Fig. 8). The data was reorded using a data logger at one second interval. To validate the experimental temperature distribution, a simulated 3D temperature distribution profile was developed using a validated heat transfer model developed by the current authors^3^. The detail of the theoretical model, from where simulation results were generated, can be found in that ref.^3^.

### NMR measurements

A Bruker DRX wide-bore spectrometer (Bruker Bio-spin, Karlsruhe, Germany) was used for NMR experiment with an operating frequency 300 MHz for hydrogen. This machine was fitted with a micro-imaging (micro 120) gradient set and a birdcage coil. During the NMR experiment, the samples were taken out of the dryer at different stages of the drying process and immediately placed in a 25 mm diameter NMR tube. There is a possibility to gain moisture from the environment while the sample is taken out from the dryer. Therefore, to protect the sample from the surrounding air, it was immersed in an inert liquid, namely *Fomblin* PFPE (Grade 06/6, USA) to provide a barrier for mass transfer between samples and surroundings. This inert liquid also can help to assist with sample shimming during NMR experimentation. Then the tube was sealed with a standard NMR tube cap and incubated at 22 °C for 5 min to reach thermal equilibrium. To collect and process the NMR data, Paravision 4 software (Bruker) was used. Following acquisition parameters: 64 averages, 1000 echoes with 10 ms echo time and 5.0 sec repetition time were used to obtain regional T_2_ relaxation times from relaxation maps acquired with a multi-slice-multi-echo (MSME) sequence. The matrix size & slice thickness were 64 × 64 and 3 mm, respectively. The spatial resolution of the scans was 468 µm. Two ROIs (region-of-interest) were defined manually from the MSME images for each sample. The mean value of these ROIs was computed for all the images. A third ROI was assessed outside the sample for determining the signal-to-noise ratio (SNR). A good SNR is needed for the accuracy of the model^22^. Therefore, the noisy T_2_-signals were eliminated from the original signal. The data were transferred to a personal computer for storage and further mathematical analysis.

### Mathematical analysis

A quantitative analysis of the water mobility in the apple tissue was undertaken by undertaking a multicomponent analysis of the T_2_ relaxation decay. A nonlinear least-squares method was applied for data analysis^46^. Generally, the free induction decay of the proton relaxation can follow an exponential decay. For multiple environments, there will be corresponding T_2_ relaxation time constants. A multi-exponential equation can describe these functions. Each tissue compartment corresponds to a different cellular environment and will have a distinct relaxation time constant (T_2_). It was assumed that these compartments were not inter-reliant at the time of the measurements; which means that the multi-exponential nature of the T_2_ decay curve relates to the different water compartments in the tissue and the water molecules do not undergo rapid exchange between compartments on the NMR time scale^4,5^. However, when water proton relaxation follows a pattern of mono-exponential decay, there is a fast exchange of protons between tissue water and macromolecules, showing that the water compartments are symbiotic^47,48^. To decide whether the signal contains one or more components, the signal can be fitted with a mono-exponential model. Fitting a mono-exponential function will result in an erroneous value for T_2_ if more than one component is present. In such cases, we need to fit for the additional component(s). The number of the component can be estimated by plotting the natural logarithm of the signal against time, after which it can be straightforward to see if the function is bi-exponential or tri-exponential, as there will be distinct linear regions of different slope in the resulting plot^49^.

In this study, after plotting the natural logarithm of the signal against time, it was decided that two significant components exist in the relaxation data. Therefore, bi-exponential fitting of T_2_ relaxation was used for this study to estimate the ICW and FW in the plant-based food material. Two components of the T_2_ relaxation curve were derived from the following expression:

1 $$Z={M}_{1}\times {\exp }^{-t/{T}_{2}^{1}}+{M}_{2}\times {\exp }^{-t/{T}_{2}^{2}}$$

where, *Z* is the function of *T*_2_ relaxation time constant, *M*_1_ and *M*_2_ represent the relative contributions of the two proton environments, and ${T}_{2}^{1}$, ${T}_{2}^{2}$ are the relaxation time constants of the different components.

The multicomponent T_2_ relaxation curve was fitted with user-defined MATLAB code (Math Works, Natick, MA). NMR sensitivity is the big limitation to analyse the T_2_ relaxation for any biological tissue. To determine the sensitivity of an NMR spectrometer, the signal-to-noise ratio measurement is an important criterion. If SNR is low, the fit to the four parameters (*M*_1_, *M*_2_, ${T}_{2}^{1}$, ${T}_{2}^{2}$) for the bi-exponential model becomes uncertain, reducing accuracy and precision. Therefore, SNR was investigated before fitting the model. To investigate the SNR, the specific borderline which can separate the signal and the noise region were selected first. Then, the SNR was calculated for all the experiments. In the present study, a SNR of 5.17 were found to be sufficient to obtain an accuracy and precision of the model^50,51^. The experiments were replicated two times for each sample, and the average of the relative contribution was taken for analysis of the results.

The detail NMR experimental procedure can be found in the authors’ previous publications^5,22^.

In addition, to confirm the investigated ICW transport phenomena are more accurate and realistic, the NMR result will be validated using another non-destructive microstructure analysis method, namely X-ray microtomography which are discussed below.

### X-ray (micro) computed tomographic experiment

X-ray microtomography is a powerful tool to analyse the microstructure of biological tissue. Extensive X-ray microtomographic experiments have been conducted at different stages of drying to validate the quantitative NMR result. Before placing into the X-ray µCT, the samples were kept in a desiccator to avoid the natural rehydration from the ambient condition. The dried samples were then scanned using a Scanco µCT high-resolution desktop µ-CT system which consists of a resolution of 6 µm. The scanning was operated at 55 KV, and the images were taken through 0°–360° of rotation. For each scan, on average, required time was 40 min. The X-ray shadow projections of the 3-D object were digitised as 2047 × 2047 pixel images, including 167 slices.

An image processing software, ImageJ (ImageJ 1.51j8, NIH, USA), was used to process and analyse the X-ray tomographic high-resolution image. Image segmentation procedure was carried out to identify the cell and cell boundary. Image segmentation is a standard process for simplifying the representation of an image into something that is more meaningful and easier to analyse. This process involves segmenting the grayscale image into a binary 8-bit image by defining the global threshold value 1 to all pixels whose intensity was below a given grey tone value and 0 to all the others^52^. In our analysis, an automated threshold on the entropy of the histogram was calculated for each image. The grayscale tone value 1 means the total transmission or reflection of light of X-ray at all visible wavelengths, resulting a white (bright) colour image. On the other hand, 0 indicates that the transmitted or reflected light of X-ray is totally absent and hence only black colour is visible. Depending on the diverse types of biological tissues, when an X-ray beam is passed to a solid matrix then it is reflected and appears white^43,53^. Based on this theory, the white colours of the analysed X-ray CT images presented in this paper indicate the cell wall of apple tissue as the cell wall is the basic building block of that tissue which is made by the solid matrix. More information about the image processing undertaken in this study can be found in authors’ previous publications^54^.

### Statistical analysis

Data are expressed as mean ± SD of the mean. For statistical analysis least-squares linear regression analysis was used. A 95% confidence level was regarded as significant.

### Code availability

The MATLAB code developed by the author can be obtained from the corresponding author upon request.

## Data Availability

All relevant data included in this article can be obtained from the corresponding author upon request.

## References

[1] Gustavsson, J., Cederberg, C. & Sonesson, U. L. F. Global food losses and food waste-Extent, causes and prevention. Food and Agriculture organization of United nations, Rome (2011).

[2] Grabowski Stefan, Ramaswamy Hosahalli, Marcotte Michele (2003). Drying of Fruits, Vegetables, and Spices. Handbook of Postharvest Technology.

[3] Khan MIH, Kumar C, Joardder MUH, Karim MA (2017). Determination of appropriate effective diffusivity for different food materials. Drying Technology.

[4] Khan, M. I. H., Wellard, R. M., Mahiuddin, M. & Karim, M. A. Cellular Level Water Distribution and Its Investigation Techniques. In Intermittent and Nonstationary Drying Technologies: Principles and Applications (pp. 193–210). CRC Press (Taylor & Francis Group) (2017).

[5] Khan MIH, Wellard RM, Nagy SA, Joardder MUH, Karim MA (2016). Investigation of bound and free water in plant-based food material using NMR T_2_ relaxometry. Innovative food Science & emerging technologies.

[6] Kumar C, Joardder MUH, Farrell TW, Karim MA (2016). Multiphase porous media model for Intermittent microwave convective drying (IMCD) of food. International Journal of Thermal Sciences.

[7] Mercier S, Marcos B, Moresoli C, Mondor M, Villeneuve S (2014). Modeling of internal moisture transport during durum wheat pasta drying. Journal of Food Engineering.

[8] Curcio S (2010). A multiphase model to analyze transport phenomena in food drying processes. Drying Technology.

[9] Nguyen TA (2006). Finite element modelling and MRI validation of 3D transient water profiles in pears during postharvest storage. Journal of the Science of Food and Agriculture.

[10] Perré, P. Multiscale aspects of heat and mass transfer during drying, in Drying of Porous Materials, Springer. 59–76 (2006).

[11] Konstankiewicz K (2002). Cell structural parameters of potato tuber tissue. International agrophysics.

[12] Khan MIH, Wellard RM, Nagy SA, Joardder MUH, Karim MA (2017). Experimental investigation of bound and free water transport process during drying of hygroscopic food material. International Journal of Thermal Sciences.

[13] Khan MIH, Nagy SA, Karim MA (2018). Transport of cellular water during drying: An understanding of cell rupturing mechanism in apple tissue. Food Research International.

[14] Bose, B. Influence of heat stress on plant responses: an approach to physico-chemical, biotechnological and molecular aspects. *Physiology of Nutrition and Environmental Stresses on Crop Productivity* 512 (2013).

[15] Ihns R (2011). Effect of temperature on the drying characteristics, colour, antioxidant and beta-carotene contents of two apricot varieties. International Journal of Food Science & Technology.

[16] Fanta SW (2013). Microscale modeling of water transport in fruit tissue. Journal of Food Engineering.

[17] Welsh Z, Simpson MJ, Khan MIH, Karim MA (2018). Multiscale Modeling for Food Drying: State of the Art. Comprehensive Reviews in Food Science and Food Safety.

[18] Aregawi WA, Abera MK, Fanta SW, Verboven P, Nicolai B (2014). Prediction of water loss and viscoelastic deformation of apple tissue using a multiscale model. Journal of physics: condensed matter.

[19] Carr EJ, Turner IW, Perre P (2013). A dual-scale modeling approach for drying hygroscopic porous media. Multiscale Modeling & Simulation.

[20] Dinçer, İ. & Zamfirescu, C. Basics of Drying. *Drying Phenomena: Theory and Applications*: p. 67–98 (2016).

[21] Prothon F, Ahrne L, Sjoholm I (2003). Mechanisms and prevention of plant tissue collapse during dehydration: a critical review. Crit Rev Food Sci Nutr.

[22] Khan MIH, Karim MA (2017). Cellular water distribution, transport, and its investigation methods for plant-based food material. Food Research International.

[23] Sedin G (2000). Lung water and proton magnetic resonance relaxation in preterm and term rabbit pups: their relation to tissue hyaluronan. Pediatric Research.

[24] Cutillo A. G., Morris A. H., Ailion D. C., Durney C. H., Ganesan K. (1992). Determination of Lung Water Content and Distribution by Nuclear Magnetic Resonance. New Aspects on Respiratory Failure.

[25] Sulyok E (2001). Brain water and proton magnetic resonance relaxation in preterm and term rabbit pups: their relation to tissue hyaluronan. Neonatology.

[26] Berenyi E (1998). Water content and proton magnetic resonance relaxation times of the brain in newborn rabbits. Pediatric Research.

[27] Vajda Z (1999). Brain adaptation to water loading in rabbits as assessed by NMR relaxometry. Pediatric Research.

[28] Inao S., Kuchiwaki H., Hirai N., Takada S., Kageyama N., Furuse M., Gonda T. (1985). Dynamics of Tissue Water Content, Free and Bound Components, Associated with Ischemic Brain Edema. Brain Edema.

[29] Furuse Masahiro, Gonda Takami, Kuchiwaki Hiroji, Hirai Nagatoshi, Inao Suguru, Kageyama Naoki (1984). Thermal Analysis on the State of Free and Bound Water in Normal and Edematous Brains. Recent Progress in the Study and Therapy of Brain Edema.

[30] Moser E, Holzmueller P, Krssak M (1996). Improved estimation of tissue hydration and bound water fraction in rat liver tissue. Magnetic Resonance Materials in Physics, Biology and Medicine.

[31] Moser E, Holzmueller P, Gomiscek G (1992). Liver tissue characterization by *in vitro* NMR: tissue handling and biological variation. Magn Reson Med.

[32] Hu Y, Wang S, Wang S, Lu X (2017). Application of nuclear magnetic resonance spectroscopy in food adulteration determination: the example of Sudan dye I in paprika powder. Scientific reports.

[33] van der Weerd L (2001). Quantitative NMR microscopy of osmotic stress responses in maize and pearl millet. Journal of Experimental Botany.

[34] Westbrook, C. & Roth, C. K. MRI in Practice. John Wiley & Sons (2011).

[35] Missbach-Guentner J (2018). 3D virtual histology of murine kidneys–high resolution visualization of pathological alterations by micro computed tomography. Scientific reports.

[36] Durrant KL, Skicko IM, Sturrock C, Mowles SL (2016). Comparative morphological trade-offs between pre-and post-copulatory sexual selection in Giant hissing cockroaches (Tribe: Gromphadorhini). Scientific reports.

[37] Chaurand P (2018). Multi-scale X-ray computed tomography to detect and localize metal-based nanomaterials in lung tissues of *in vivo* exposed mice. Scientific reports.

[38] Moosmann J (2013). X-ray phase-contrast *in vivo* microtomography probes new aspects of Xenopus gastrulation. Nature.

[39] Diels E (2017). Assessment of bruise volumes in apples using X-ray computed tomography. Postharvest Biology and Technology.

[40] Si Y, Sankaran S (2016). Computed tomography imaging-based bitter pit evaluation in apples. Biosystems Engineering.

[41] Madiouli J (2011). Non-contact measurement of the shrinkage and calculation of porosity during the drying of banana. Drying Technology.

[42] Cantre D, Herremans E, Verboven P, Ampofo-Asiama J, Nicolaï B (2014). Characterization of the 3-D microstructure of mango (Mangifera indica L. cv. Carabao) during ripening using X-ray computed microtomography. Innovative Food Science & Emerging Technologies.

[43] Léonard, A., Crine, M. & Stepanek, F. Use of X-ray tomography for drying-related applications. In: Tsotsas, E. & Mujumdar, A. S., editors vol. 2, Weinheim: Wiley-VCH Verlag GmbH; p. 143–86 (2009).

[44] Mahiuddin M, Khan MIH, Pham ND, Karim MA (2018). Development of fractional viscoelastic model for characterizing viscoelastic properties of food material during drying. Food bioscience.

[45] Mahiuddin M, Khan MIH, Kumar C, Rahman MM, Karim MA (2018). Shrinkage of food materials during drying: Current status and challenges. Comprehensive Reviews in Food Science and Food Safety.

[46] Mulkern RV (1989). Two-site exchange revisited: a new method for extracting exchange parameters in biological systems. Biophysical Journal.

[47] Cole WC, LeBlanc AD, Jhingran SG (1993). The origin of biexponential T2 relaxation in muscle water. Magnetic Resonance Med.

[48] Shioya S (1990). A 1-year time course study of the relaxation times and histology for irradiated rat lungs. Magn Reson Med.

[49] Momot KI, Pope JM, Wellard RM (2010). Anisotropy of spin relaxation of water protons in cartilage and tendon. NMR Biomed.

[50] Boulby, P. A. & Rugg-Gunn, F. T2: the transverse relaxation time. Quantitative MRI of the brain. Wiley, Chichester 143–202 (2003).

[51] Wheeler-Kingshott, C. A. M. *et al*. D: The Diffusion of Water, in Quantitative MRI of the Brain. John Wiley & Sons, Ltd. p. 203–256 (2004).

[52] Laverse J (2012). X-ray microtomography to study the microstructure of mayonnaise. Journal of Food Engineering.

[53] Seleþchi, E. & Duliu, O. G. Image processing and data analysis in computed tomography. 7^th^ International Balkan Workshop on Applied Physics, 5–7, Constanta, Romania (July 2006).

[54] Rahman MM, Gu YT, Karim MA (2018). Development of Realistic Food Microstructure Considering the Structural Heterogeneity of Cells and Intercellular Space. Food Structure.

